# A Clinical Outcome of the Anti-PD-1 Therapy of Melanoma in Polish Patients Is Mediated by Population-Specific Gut Microbiome Composition

**DOI:** 10.3390/cancers14215369

**Published:** 2022-10-31

**Authors:** Bernadeta Pietrzak, Katarzyna Tomela, Agnieszka Olejnik-Schmidt, Łukasz Galus, Jacek Mackiewicz, Mariusz Kaczmarek, Andrzej Mackiewicz, Marcin Schmidt

**Affiliations:** 1 Department of Biotechnology and Food Microbiology, Poznan University of Life Sciences, 60-627 Poznan, Poland; 2 Department of Cancer Immunology, Chair of Medical Biotechnology, Poznan University of Medical Sciences, 60-627 Poznan, Poland; 3 Department of Medical and Experimental Oncology, Poznan University of Medical Sciences, 60-627 Poznan, Poland; 4 Department of Cancer Diagnostics and Immunology, Greater Poland Cancer Centre, 60-627 Poznan, Poland

**Keywords:** microbiome, melanoma, immune checkpoint inhibitor, diet, immunotherapy

## Abstract

**Simple Summary:**

The gut microbiome develops rapidly after birth and is constantly modified by many factors, especially, lifestyle, diet, and drugs. It affects the performance of the immune system, and it also impacts the response to immunotherapy. This article presents the association between the response to anti-PD-1 therapy and the baseline gut microbiome, alongside selected agents modifying the microbiome composition in a Polish cohort of melanoma patients.

**Abstract:**

The gut microbiota is considered a key player modulating the efficacy of immune checkpoint inhibitor therapy. The study investigated the association between the response to anti-PD-1 therapy and the baseline gut microbiome in a Polish cohort of melanoma patients, alongside selected agents modifying the microbiome. Sixty-four melanoma patients enrolled for the anti-PD-1 therapy, and ten healthy subjects were recruited. The response to the treatment was assessed according to the response evaluation criteria in solid tumors, and patients were classified as responders or non-responders. The association between selected extrinsic factors and response was investigated using questionnaire-based analysis and the metataxonomics of the microbiota. In the responders, the Bacteroidota to Firmicutes ratio was higher, and the richness was decreased. The abundance of *Prevotella* *copri* and *Bacteroides* *uniformis* was related to the response, whereas the non-responders’ gut microbiota was enriched with *Faecalibacterium* *prausnitzii* and *Desulfovibrio* *intestinalis* and some unclassified Firmicutes. Dietary patterns, including plant, dairy, and fat consumption as well as gastrointestinal tract functioning were significantly associated with the therapeutic effects of the therapy. The specific gut microbiota along with diet were found to be associated with the response to the therapy in the population of melanoma patients.

## 1. Introduction

Immune checkpoint inhibitor (ICI) therapy has revolutionized oncology and become the major approach to fight various cancers, including melanoma [1]. The ICI mechanism relies on blocking the immune checkpoints or their ligands through specific antibodies to prevent their binding and enhance the anticancer responses. Cytotoxic T lymphocyte-associated molecule-4 (CTLA-4) or programmed cell death receptor-1 (PD-1) and its major ligand—programmed cell death receptor-1 ligand (PD-L1) are the best-described targets for ICI therapy [2,3].

Despite the notable and long-lasting benefits from ICI therapy observed in melanoma patients, up to 65% and more than 70% of patients receiving PD-1 and CTLA-4 inhibitors, respectively, did not respond to therapy due to primary resistance, and one-third of initially responding patients developed drug resistance and melanoma progression within 3 years [4].

Several tumor- and host-associated agents are considered predictive biomarkers for response to the treatment, and their correlation with ICI therapy’s efficacy was broadly described in our previous paper [5]. Numerous studies have consistently demonstrated the association of the gut microbiota composition with the response and survival benefits from ICI therapy, mostly manifested as progression-free survival (PFS) or overall survival (OS); however, there were contradictions in the specific taxa identified as positively or negatively associated with the clinical outcomes between various cohorts [6]. A recent study demonstrated that this link is cohort-dependent, and the presence or absence of a single species cannot serve as a predictive biomarker across various populations [7]. Moreover, several factors, such as diet or concomitant drugs [8,9], were associated with changes in the gut microbiota composition and immune system functions, and their role in ICI efficacy modulation has yet to be fully elucidated.

The primary goal of our study was to investigate the association between the response to anti-PD-1 therapy and the baseline gut microbiome, alongside selected agents modifying microbial composition in the intestines in a cohort of melanoma patients. To our knowledge, this is the first study on the Polish population analyzing this aspect, and we believe that the present study will give new insight into the complex relationship between the gut microbiome and ICI treatment efficacy.

## 2. Materials and Methods

### 2.1. Study Cohort and Data Collection

Patients with histologically confirmed unresectable stage III or stage IV cutaneous melanoma enrolled in treatment with anti-PD-1 therapy (nivolumab or pembrolizumab), as a part of the Ministry of Health (Poland) drug program [10], were recruited to the study at the Department of Medical and Experimental Oncology, Heliodor Święcicki Clinical Hospital, Poznan University of Medical Sciences (Poznan, Poland), from June 2018 to December 2021. Furthermore, we also recruited healthy subjects, who served as a control group. They were patients’ close relatives living in the same household. The enrolled cohort comprised 64 melanoma patients and 10 healthy subjects. Written informed consent was obtained from all participants. The study was approved by the Bioethics Committee at Poznan University of Medical Sciences (registration number 402/18).

Clinical information including tumor stage and serum lactate dehydrogenase (LDH) concentration was collected from the medical records. Response to ICI therapy was assessed according to the response evaluation criteria in solid tumors (RECIST) v.1.1 criteria. On that basis, responders (R) were defined as patients with complete (CR) or partial (PR) response, while non-responders (NR) were defined as patients with disease stabilization (SD) or progression (PD). Additional classification categorized patients into those who exhibited clinical benefit (CB) from ICI therapy (CR, PR, and SD) and those who did not (PD) abbreviated as ‘NB’.

### 2.2. Questionnaire

All study participants were asked to fill out anonymized questionnaire (included in supplemental information Document S1) during their visit to the hospital and return it to the medical staff, who did not intervene in the survey completion. The questionnaires were collected before starting the anti-PD-1 therapy, consisted of 39 closed questions, and covered socio-demographic, anthropometric, clinical, lifestyle, and dietary variables. Statistical analysis of the data obtained from the questionnaires was performed in R language environment v. 4.1.3. The Kruskal–Wallis test (for numerical data) and Fisher’s exact test (for categorical data) were used to study whether there were significant differences between responders and non-responders in terms of variables, accepting a confidence level of 95%. Subsequently, the relative risk (RR) is defined as the probability of reaching the outcome in the presence of the risk factor, divided by the probability of reaching the outcome in the absence of this risk factor, which was calculated for selected variables.

### 2.3. Fecal Sample Collection and Stool Calprotectin Measurement

Fecal samples were collected from melanoma patients before ICI treatment commenced and from healthy subjects. All participants received oral and written instructions regarding the stool-collection procedure (according to the modified protocol described by Dore et al. (2015) [11]). Briefly, they were requested to collect ~30 g of feces into provided fecal containers, preferably on the day of the scheduled hospital visit, and then store the samples at room temperature or 2–8 °C until they arrived at the hospital. The stool samples were delivered to the laboratory at room temperature, and 1 g of each stool sample was placed into a tube with RNAlater and incubated for 1 h at room temperature. Afterward, RNAlater-preserved feces were homogenized and centrifuged at 10,500 RPM for 3 min, and then the supernatant was thoroughly removed. Approximately 250 mg of fecal slurry was aliquoted into tubes and frozen at −20 °C until metagenomic DNA extraction.

Additionally, 15 mg of raw fecal samples was collected, and calprotectin concentration in stool was measured with IDK Calprotectin ELISA Kit (Immundiagnostik AG, Bensheim, Germany), according with the instructions of the kit manual.

### 2.4. Metagenomic DNA Extraction and Metagenome Sequencing

Metagenomic DNA was extracted from approximately 250 mg of RNAlater-preserved fecal slurry, using commercial system DNeasy PowerSoil Pro (QIAGEN), in accordance with the protocol of the manufacturer. With an additional step of RNase digestion (50 µg per sample, 10 min, 60 °C) after the bead-beating step. The DNA concentration in the samples was determined fluorometrically using the QuantiFluor dsDNA System (Promega, Madison, WI, USA), in accordance with the instructions of the kit manual. All DNA samples were diluted to a concentration of ~5 ng µL^−1^ before sequencing.

For 16S-targeted metagenome sequencing, the V3-V4 hypervariable regions of the 16S rRNA gene were amplified using 341F (5′-TCG TCG GCA GCG TCA GAT GTG TAT AAG AGA CAG CCT ACG GGN GGC WGC AG-3′) and 785R (5′-GTC TCG TGG GCT CGG AGA TGT GTA TAA GAG ACA GGA CTA CHV GGG TAT CTA ATC C-3′) primers [12] and KAPA HiFi HotStart Ready Mix (Roche, Basel, Switzerland). The NEBNext Ultra DNA library for Illumina (New England BioLabs, Ipswich, MA, USA) was used to generate sequencing libraries, in accordance with the recommendations of the manufacturer. The library quality check was performed with a Qubit 2.0 Fluorometer (Thermo Scientific, Waltham, MA, USA) and Agilent Bioanalyzer 2100 system. The sequencing was carried out on Illumina MiSeq PE300. Both second stage of library preparation and metagenome sequencing was done by Genomed S.A. (Warsaw, Poland). The 16S rRNA gene sequences undergone initial processing using the DADA2 v. 1.21.0 package in the R language environment v. 4.1.3. Forward reads were truncated at 275 bp, and reverse reads were truncated at 220 bp, before merging. Then, chimeric sequences were removed, and taxonomy assigned to each merged sequence, referred to as amplicon sequence variants (ASVs) at 100% similarity, using the SILVA SSU database release 138 [13]. The alpha- and beta-diversity measures were calculated in the R using the Microbiome 1.13.8, Phyloseq 1.34.0, and vegan 2.5.7 packages.

Shotgun library preparation and metagenome sequencing were performed by Genomed S.A. (Warsaw, Poland). For library preparation, 250 ng DNA per sample fragmented in E220 Focused-ultrasonicator (Covaris LLC., Woburn, MA, USA) was used with NEBNext Ultra II DNA Library Prep Kit for Illumina (New England Biolabs, Ipswich, MA, USA), in accordance with the guidelines of the manufacturer. Sequencing was performed using Illumina NovaSeq 6000 PE150. The quality of data received from the sequencing company was checked with FastQC (mean quality score > 35). All samples were pre-filtered, and adapters were removed by the sequencing company. The sequences were further analyzed with Genome Extraction from Shotgun Metagenome Sequence Data workflow [14] on the KBase.us server [15].

## 3. Results

### 3.1. Characteristics of the Study Cohort

In total, we recruited 64 patients with unresectable stage III or stage IV cutaneous melanoma, eligible for ICI treatment using the anti-PD-1 molecules (nivolumab or pembrolizumab), and 10 healthy subjects, who served as controls. The questionnaires and stool samples were collected from the enrolled patients before (at baseline) the initiation of the anti-PD-1 infusion and from the healthy subjects, who accompanied the patients during their visit to the hospital.

The baseline socio-demographic and clinical characteristics of patients and controls are shown in Table 1. Sex distribution was comparable between groups (*p* > 0.05). Statistical analysis showed that the control group consisted of significantly younger individuals than the patient group (*p* < 0.05). The distribution of the metastatic stage did not differ in both groups (*p* > 0.05). Moreover, the concentration of serum LDH that qualified as elevated (>250 U/L) was observed in 21% of the responders and 42% of the non-responders. Although no statistically significant differences (*p* > 0.05) in the normal and elevated serum LDH level distribution between the responders and non-responders were found to be qualified according to Deckers et al. [16], the median value of the marker was significantly higher (*p* < 0.05) in the non-responders compared to the responders.

### 3.2. Baseline Gut Microbiota- and Immune Response-Modifying Agents Significance in the Response to Anti-PD-1 Therapy in the Cohort of Melanoma Patients

The questionnaire-based analysis revealed statistically significant differences between the patient groups. The data collected from the returned questionnaires are summarized in Table 2.

There was a significantly lower frequency of Rh-positive and a higher frequency of Rh-negative blood type patients, showing the progression of the disease (*p* < 0.05), compared to those with clinical benefit.

Furthermore, differences in baseline dietary patterns among the patients undergoing anti-PD-1 therapy were also taken into consideration. Analysis of the prevailing fat type in the patients’ diet showed statistically significant differences between the responders and non-responders (*p* < 0.05). The most pronounced difference between the patient groups was found in terms of both the plant- and animal-based (mixed) dietary fat consumption, as 36.0% of the responders declared consumption of mixed dietary fats, whereas 6.9% of the non-responders followed a similar dietary pattern. Furthermore, prevailing plant-based meal consumption was also found to be significantly associated with the response to antiPD-1 therapy (*p* < 0.05). Plant-based meal consumption was assessed as low or high and consisted of pooled categories of fruit and vegetable portions consumed daily. Participants who declared a diet change were excluded from the analysis; however, the vast majority of participants, 95.8% of the responders, 86.2% of the non-responders, and 80.0% of the controls, had not changed their diet recently. We found that the high consumption of plant portions was associated with a 2.9-fold increase in the probability of responding to anti-PD-1 therapy. However, when we analyzed vegetable and fruit portion consumption independently, we did not find any statistically significant differences between the studied groups (*p* > 0.05). Moreover, an analysis of dairy portion consumption that included the most numerous cohorts, i.e., those who declared the consumption of either 1 or fewer or 2–3 dairy portions daily, showed significant differences between the responders and non-responders (*p* = 0.05) and between CB and NB (*p* < 0.05). We found that low consumption (1 or fewer daily) of dairy portions was associated with a 2.0-fold increase in the probability of responding to anti-PD-1 therapy.

Additionally, significant differences between the responders versus the non-responders and between the patients versus the controls were found (*p* < 0.05), regarding baseline defecation frequency. We observed that regular once-a-day defecation was a practice of 62.5% of the responders, 43.3% of the non-responders, and 50.0% of the controls. Additionally, 40.0% of the non-responders, 4.2% of the responders, and 20.0% of the controls defecated every second day.

The other investigated factors were not found to be significantly associated with the clinical outcome to anti-PD-1 therapy in the melanoma patient cohort.

Moreover, a borderline significance was found, in terms of the birthplace, between the controls and patients (*p* = 0.058). Briefly, 70.0% of the controls and 54.9% of the patients were born in a hospital.

### 3.3. Baseline Gut Microbiota of Responders Was Enriched with Prevotella copri and Bacteroides Uniformis

The analysis of the gut microbiota composition included 25 responders, 32 non-responders, and 10 controls.

**Figure 1:**
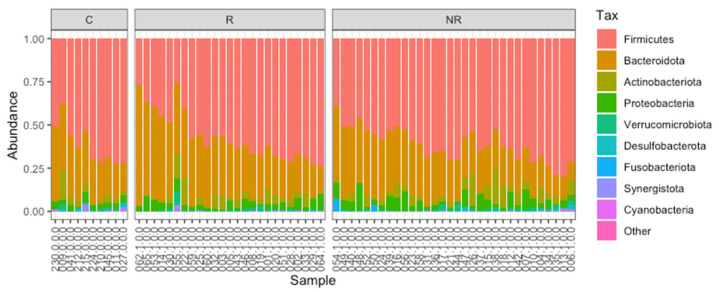
The gut microbiota composition (relative abundance) of the study cohort at the phylum level. Analyzed samples were grouped into C—controls, R—responders, and NR—non-responders.

**Figure 2:**
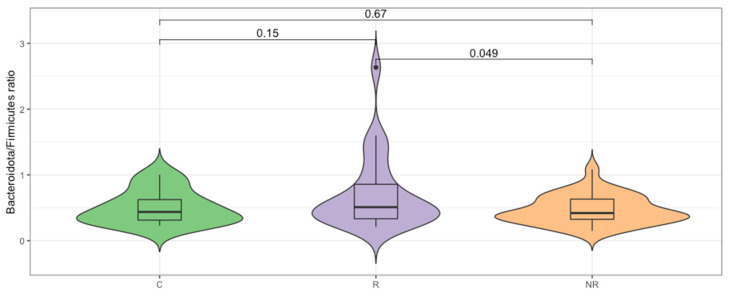
Bacteroidota/Firmicutes ratio differs significantly (*p* < 0.05, *t*-test) for the responder (R) and non-responder (NR) groups.

Firmicutes and Bacteroidota were found to be the most abundant taxa at the phylum level in the responders, non-responders, and controls (Figure 1). The median Bacteroidota to Firmicutes ratio was higher in the responder group (0.51) than in the control group (0.44), whereas it was lower in the non-responder group (0.42). The difference between the R–NR groups was statistically significant (*p* < 0.05, Figure 2). The commensal microbiota modulates inflammatory/immune responses in the organism and is, therefore, likely to play a major role in regulating inflammation and immunity to cancer at different levels [17]. 

**Figure 3:**
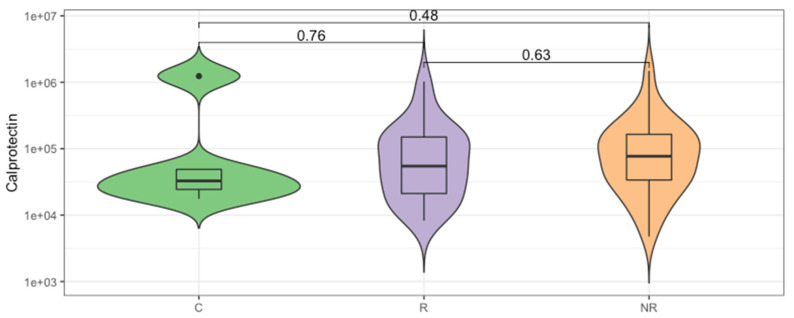
The inflammatory state of gut mucosa was evaluated by calprotectin concentration measurement in the stool of study participants. Comparison between groups of controls (C) and the anti-PD-1 recipients before the first injection of the ICI therapeutics, classified as responders (R) and non-responders (NR) according to the clinical outcome of ICI therapy. The *p*-values describing the statistical significance of the calprotectin level in the study groups were calculated with Wilcoxon Rank Sum Test. The plot is in a logarithmic scale.

The enrichment in Bacteroidota can lead to a reduction in intestinal inflammation [18,19,20]. Therefore, we analyzed the concentration of fecal calprotectin, which is an intestinal inflammatory marker. The median calprotectin concentration in the stool was 32.75, 54.69, and 76.99 µg/mL, respectively, for the control, responder, and non-responder groups (Figure 3), which indicates lower inflammatory responses in the responder group and is in agreement with the B/F ratio. However, the differences in the calprotectin concentration between the study groups were not statistically significant (*p* > 0.05).

**Figure 4:**
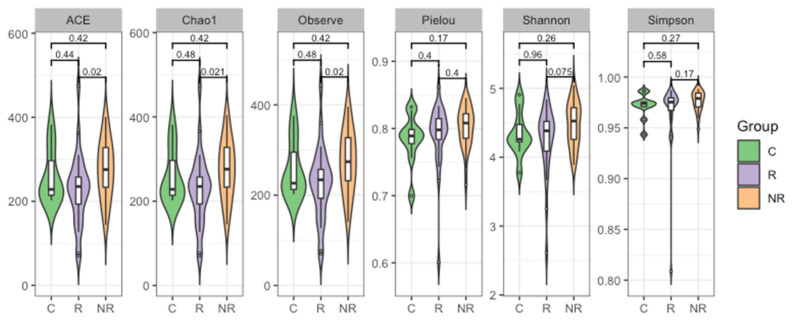
Plots depicting a comparison of bacterial ASV alpha diversity of the stool microbiota as estimated regarding richness (ACE, Chao1, and observed indices), evenness (Pielou index), and diversity (Shannon and Simpson indices) measures in study groups (R—responders, NR—non-responders, and C—controls). Wilcoxon Rank Sum Test indicated that the responder and non-responder groups’ microbiota differed in regards to richness (*p* < 0.05).

Furthermore, the alpha and beta diversity of the gut microbiota among the studied groups were analyzed. Alpha diversity is a measure of the compositional complexity of a community within a sample. Its value increases with the number of identified species and with the evenness of their relative abundances. Richness indices evaluate the number of different species in a sample; evenness indices weigh up the species’ relative abundances, without focusing on their total number; and diversity indices consider both the species’ relative abundances and the total number of different species [21]. We found that the gut microbial community of the patients responding to anti-PD-1 therapy was significantly less rich in bacterial species than that of the non-responders (*p* < 0.05, Figure 4).

**Figure 5:**
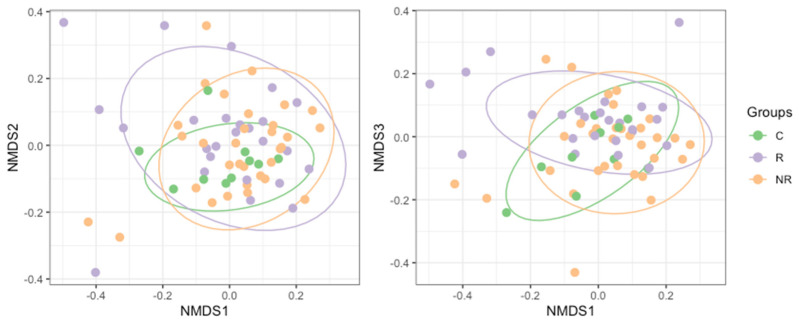
NMDS (non-metric multidimensional scaling) plot based on Bray–Curtis dissimilarity of the bacterial microbiota composition in study groups (R—responders, NR—non-responders, and C—controls). Permutation multivariate analysis of variance revealed significant differences between the groups (R^2^ = 0.0491 and *p* = 0.0089).

Differences between the bacterial microbiota composition (beta diversity) in the study groups were examined using non-metric multidimensional scaling (NMDS, Figure 5). We found that the baseline gut microbiota of the patients responding to anti-PD-1 therapy was significantly different from that of the non-responders’ (R^2^ = 0.0357, *p* = 0.0033), as determined with permutational multivariate analysis of variance (PERMANOVA). By applying a two-group comparison at the community level with a linear model [22] (limma package [23]), we were able to identify the most significantly different taxa between the responders and non-responders. The analysis revealed a strong association between the response and enrichment in the ASVs belonging to *Prevotella copri* and *Bacteroides uniformis*. However, ASVs that were unidentified to species level, belonging to orders *Izemoplasmatales* and *Clostridia* UCG-014, to family *Oscillospiraceae*, and to species *Faecalibacterium prausnitzii* and *Desulfovibrio intestinalis*, were significantly enriched in the non-responders (Table 3).

Furthermore, we performed shotgun sequencing of selected gut microbiota samples (*n* = 16), which resulted in an average of 41.37 million high-quality reads and 40.24 million reads of 145–151 bp read-size distributions. The received sequences were pre-filtered, had adapters removed by the sequencing company, and were further evaluated with FastQC software [24]. The adapter content was lower than 0.1%, and the mean quality score was >35. The high-quality filtered paired-end reads were assembled into contigs (Table S1) using metaSPAdes v3.15.3 [25] and MEGAHIT v1.2.9 [26], which resulted in an average of 29.25 thousand contigs per sample. For further processing, the better assembly was used as determined by the Compare Assembled Contig Distributions v1.1.2 application [15]. The assembled metagenomic contigs were clustered into bins, each of which corresponds to a putative population genome with MaxBin2 v2.2.4 [27], CONCOCT v1.1 [28], and MetaBAT2 v1.7 [29]. The bacterial and archaeal binned contigs from the above-mentioned binning algorithms were integrated to calculate an optimized, non-redundant set of bins from a single assembly with DAS Tool v1.1.2 [30]. An average of 7.69 thousand binned contigs resulted in the assembly of an average of 59 bins per sample. The binned contigs underwent further quality assessment with CheckM v1.0.18 [31] and were filtered for >90% completeness and <5% contamination. The filtered bins were annotated with RASTtk v1.073 [32] and classified with GTDB-Tk v1.7.0 [33] to obtain reconstructed metagenome-assembled genomes (MAGs). Out of 631 reconstructed MAGs in total, 19 were classified to the *Prevotellaceae* family and 15 to *Prevotella* spp., among which only 2 were *P. copri*. Recent findings indicated that *P. copri* is not a monotypic species but is composed of four distinct clades [34], so we decided to determine the clade to which our MAGs belong. A comparison of all 19 *Prevotellaceae* MAGs, with 20 selected exemplars, curated *P. copri* MAGs (Table S2) with dRep v3.1.0 [35] and revealed another member of *P. copri*, previously classified as *Prevotella* sp900313215 by GTDB-Tk, as determined by MASH Average Nucleotide Identity [36]. Among three *P. copri* MAGs, two belonged to clade A and one to clade B (Figure S1).

## 4. Discussion

Here, we present the prospective cohort study with the main goal of identifying the association of the baseline gut microbiome and the selected factors affecting the gut microbiota and/or immune responses, with the therapeutic effectiveness of ICI therapy in the cohort of metastatic melanoma patients.

We analyzed the baseline gut microbiota composition by targeted NGS, as the immunomodulatory effects of the gut microbiota and microbial metabolites are well-established, and numerous studies have demonstrated remarkable differences in the gut microbiota composition between various cancer patients, responding and non-responding to ICI therapy, indicating its impact on treatment efficacy [6]. Our analysis included fecal samples derived from 25 responders, 32 non-responders, and 10 controls. In our cohort, the baseline gut microbiota was dominated by Firmicutes and Bacteroidota phyla (Figure 1). However, the median Bacteroidota to Firmicutes ratio was significantly higher in the responders than in the non-responders (Figure 2), indicating that there was an increased relative abundance of Bacteroidota to Firmicutes in the responders compared to the non-responders. Contradictory findings were demonstrated by Chaput et al. (2017), who found that patients whose baseline gut microbiota was enriched with *Faecalibacterium* and other Firmicutes benefited more from the anti-CTLA-4 therapy (manifested as longer PFS and OS) than those with *Bacteroides*-driven microbiota [37].

The Bacteroidota to Firmicutes ratio is considered a marker of homeostasis in the intestines, and any alterations in the relative abundance may lead to the development of various diseases. Overall, studies reported that an increase in the abundance of specific Firmicutes species is associated with obesity, due to their carbohydrate and lipid fermentation and metabolism properties. Whereas, Bacteroidota species were found to exert pro-inflammatory responses that correlated with inflammatory bowel disease (IBD) development [38]. However, both Firmicutes and Bacteroidota are large phyla that cover numerous species with various properties. For instance, the gut microbiota enrichment with *Bacteroides* species can lead to a reduction in intestinal inflammation, as *B. fragilis* induces regulatory T cells to produce anti-inflammatory interleukin (IL)-10 inside the gut [18]. Another study demonstrated the anti-inflammatory and epithelium-reinforcing properties of *Bacteroides* and *Parabacteroides* spp. [39]. In fact, the fecal calprotectin concentration at the baseline revealed that the intestinal inflammation in the responders was lower than in the non-responders; however, the difference was not statistically significant (Figure 3). A comparable correlation between low baseline intestinal inflammation and disease control was demonstrated in patients with hepatocellular carcinoma (HCC) treated with the CTLA-4 and/or PD-L1 inhibitors, suggesting that durable inflammation in the gut may lead to the exhaustion of the immune system and, in consequence, facilitate tumor immune escape [40]. It is worth noting that there are age-related changes in fecal calprotectin concentration, and the lowest median calprotectin level was observed in the controls (Figure 3) that were significantly younger than the patients (Table 1); this result is in a line with previous studies [41].

In our cohort, the richness, evenness, and diversity of the gut microbiota were decreased in the responders compared to the non-responders. However, the significance of the difference was reached between the responders and non-responders only regarding richness (Figure 4). In contrast, several studies reported that high gut microbiota richness and diversity correlated with response and survival benefits from ICI therapy in various cohorts of cancer patients [42,43,44,45]. In addition, significant compositional differences in the gut microbiota (beta diversity) were found between the responders, non-responders, and controls (Figure 5).

Furthermore, the abundance of *Prevotella copri* and *Bacteroides uniformis* was related to the response to anti-PD-1 therapy in our cohort of melanoma patients (Table 3). Jin et al. (2019) also demonstrated that the gut microbiota of Chinese patients with advanced non-small cell lung cancer (NSCLC) responding to anti-PD-1 therapy was enriched with *P. copri* [45]. Studies demonstrated that *P. copri* is more abundant in rural and isolated (non-Westernized) populations that follow traditional lifestyles and plant-based diets compared to industrialized (Westernized) populations [46]. In our study, there was a significantly higher consumption of plants (pooled fruit and vegetable portions) in the responders compared to the non-responders (Table 2). However, *P. copri* was found to induce proinflammatory responses that may lead to the development of several diseases [47]. For instance, the abundance of *P. copri* in the intestinal microbiota correlated with rheumatoid arthritis [48,49,50], colon dysbiosis in HIV-infected subjects [51], and insulin resistance [52]. In contrast, another study indicated that *P. copri* was associated with improved glucose metabolism in subjects on a fiber-rich diet [53]. The discrepancies presented in the previous studies may be related to the heterogeneity within *P. copri* species; however, further research should be done to investigate this aspect. Tett et al. (2019) revealed that *P. copri* is composed of four distinct clades with diversified functional properties that are frequently co-present within non-Westernized individuals [34]. Our research indicated the presence of *P. copri* belonging to clades A and B in our cohort of melanoma patients (Figure S1). Clade A is the most prevalent (91.5% in non-Westernized populations versus 26.9% in Westernized populations), and clade B is the most genetically divergent from the other clades, with genetic distances between clades (interclade) of approximately 20% shown as pairwise average nucleotide identity distances (ANI distance). Moreover, the four *P. copri* complex clades show distinct carbohydrate metabolism repertoires [34] that may provide an advantage only in the case of a plant-rich diet [53].

Numerous studies demonstrated that ICI efficacy was associated with an abundance of *Bacteroides* species. For instance, baseline gut microbiota enrichment with *Bacteroides* was associated with shorter PFS and OS in melanoma patients treated with CTLA-4 inhibitors [37]. Comparably, the abundance of *B. ovatus*, *B. dorei*, and *B. massiliensis* was found to be related to poor clinical outcomes in another cohort of melanoma patients undergoing ICI therapy [44]. In contrast, it was also demonstrated that *B. thetaiotaomicron* and *B. fragilis* improved the CTLA-4 inhibitor efficacy [54], and *B. caccae* and *B. thetaiotaomicron* correlated with the response to ICIs [55]. In our cohort, enrichment with *B. uniformis* was significantly associated with response to the anti-PD-1 therapy (Table 3). Overall, immunomodulatory properties of *B. uniformis* were reported. For instance, the administration of *B. uniformis* improved the metabolic and immune functions in mice with diet-induced obesity [56,57,58]. Moreover, in vitro studies demonstrated that *B. uniformis* reduced the lipopolysaccharide-induced release of interleukin 8 (IL-8) and enhanced the integrity of enterocytes [39]. In addition, decreased glycosaminoglycan metabolism, associated with depletion of *B. uniformis*, promoted rheumatoid arthritis and osteoarthritis [59].

Furthermore, the abundance of *F. prausnitzii*, *D. intestinalis* and some unclassified Firmicutes were associated with disease stabilization or progression in our study (Table 3). In contrast, numerous studies indicated that the gut microbiota enrichment with *Faecalibacterium* spp. was associated with the response and prolonged clinical benefits from ICI therapy in various cohorts of melanoma patients [37,42,44,55,60]. Chaput et al. (2017) demonstrated that melanoma patients, whose baseline gut microbiota was driven by *Faecalibacterium* spp., had a lower frequency of regulatory T cells (Tregs) and α4^+^β7^+^CD4^+^ and α4^+^β7^+^CD8^+^ T cells before the anti-CTLA-4 therapy commencement compared to those with *Bacteroides*-driven gut microbiota, which was also associated with clinical benefit [37]. Another study revealed that *Faecalibacterium* spp. abundance correlated with higher levels of tumor-infiltrating CD8^+^ T cells and effector CD4^+^ and CD8^+^ T cells in the systemic circulation and peripheral cytokines that promoted a response to anti-PD-1 therapy, and these markers were inverse in those with *Bacteroidota* abundance [42]. Interestingly, another study indicated that *F. prausnitzii* abundance was negatively associated with the serum butyrate concentration that was found to reduce anti-CTLA-4 therapy efficacy [60].

Another species enriched in the non-responders was *D. intestinalis*, which was identified in humans [61] and animals [62,63]; however, little is known about the function of *D. intestinalis* in the human gut. The bacterium produces hydrogen sulfide (H_2_S), an endogenous signaling gasotransmitter with both pro- and anti-inflammatory properties [64]. Overall, *D. intestinalis* belongs to the sulfate-reducing bacteria, and their increased abundance was found to be associated with IBD development [65,66]. Nevertheless, *Desulfovibrio* is associated with healthy hosts in some human populations [67].

A recent study conducted on several cohorts of melanoma patients treated with ICI therapies demonstrated that the association between the gut microbiome and the response to ICIs is cohort-dependent, and the microbiome-based signatures are not consistent across cohorts [7]; therefore, our data provide an important supplement to the overall information in the field.

Furthermore, in the questionnaire-based analysis, we investigated the influence of several factors that were previously reported to affect clinical outcomes and/or gut microbiota composition, such as perinatal factors, BMI, diet, lifestyle habits, or medication, in response to anti-PD-1 therapy in our cohort of melanoma patients.

A borderline higher frequency of individuals born in hospitals versus at home was observed in the control group compared to the patients (Table 2). We suggest that this discrepancy is associated with the changes in the approach to delivery that occurred in Poland in the 1960s, when most deliveries started to take place in hospitals [68]. The controls were significantly younger than the patients, and the median age of controls was 52.5, which corresponds with the time of such changes in approach to deliveries in Poland (Table 1).

Moreover, we found a significantly higher frequency of the Rh-positive blood-type patients in the group, who benefited from the anti-PD-1 therapy, versus those with disease progression (Table 2); however, we did not find any study that demonstrated a similar correlation.

Analysis of dietary patterns demonstrated several differences between the responders and non-responders. Firstly, we found a correlation between dietary fat type and response to anti-PD-1 therapy (Table 2). Our analysis did not indicate, however, which type of fat prevailing in diet would be associated with a response to treatment; in addition, the responders more frequently consumed mixed (both plant- and animal-based) dietary fat types than the non-responders. In the questionnaire, the prevailing dietary fat type was categorized as animal-based or plant-based; therefore, we did not have a broad insight into the range of products that were consumed by patients, especially those who declared consumption of the mixed dietary fat type. However, the impact of dietary fats on clinical outcomes of ICI therapy should be further investigated, as dietary fatty acids were shown to affect the functions of both innate and adaptive immune responses through multiple pathways. Their influence may depend on the balance between the n-6 and n-3 polyunsaturated fatty acids, diet diversity, or microbiome [69]. To our knowledge, an analysis of the direct association between dietary fat consumption and the clinical outcomes of ICI therapy has not been performed yet. However, polyunsaturated fatty acids were found to exert effects that, potentially, would enhance immunotherapy efficacy [70].

We also found that high plant consumption was significantly associated with the improved clinical outcomes of anti-PD-1 therapy in our cohort (Table 2). Patients consuming high portions of plants gained a 2.9-fold increase in the possibility of response to the treatment. Various components included in fruits and vegetables, i.e., fiber, folate, vitamins, and non-nutrient phytochemicals, such as carotenoids and flavonoids, which have a profound impact on the immune system, may potentially also affect the clinical outcomes of ICI therapy [71]. A recent study demonstrated that sufficient dietary fiber intake (≥20 g/day) improved ICI therapy efficacy in melanoma patients [72]. This effect was corroborated in conventionally housed, but not germ-free, mice fed with a fiber-rich diet, which suggests the involvement of the gut microbiota in antitumor response, and differences in the gut microbiota of mice on a fiber-rich versus a fiber-low diet were found. Moreover, mice on a fiber-rich diet had a significantly higher level of stool propionate, but not total stool short-chain fatty acid (SCFA) levels, and a significantly higher antitumor T cell response. SCFAs, such as acetate, propionate, and butyrate, are products of microbial conversion of ingested dietary fiber and mucosal glycans and exert an impact on immune responses [73]. Several studies reported that high fecal and plasma SCFA levels correlated with the improved clinical outcomes to anti-PD-1 therapy [74,75]. In contrast, another study demonstrated that high plasma SCFA levels were associated with shorter PFS in cancer patients treated with CTLA-4 inhibitors [60]. High plant consumption may correlate with increased dietary fiber intake not only because of fruits and vegetables but also because cereals and nuts are known as the main sources of dietary fiber [76]. Analysis of other fiber-rich product consumption in our study revealed that pooled plants, but not fruit, vegetable, bread type, and cereal consumption, analyzed independently was significantly associated with response to the anti-PD-1 therapy (Table 2).

Our data indicated that the patients consuming low dairy portions (1 or fewer per day) gained a 2.0-fold increase in the probability of responding to anti-PD-1 therapy compared to those who consumed high dairy portions (from 2 to 3 per day) in our cohort (Table 2). To our knowledge, the association between dairy product consumption and ICI efficacy has not been elucidated. Previous studies demonstrated contradictory findings in terms of the correlation between dairy products’ consumption and obesity [77], type 2 diabetes [78], or cardiovascular disease development [79]. Moreover, several studies presented the association between dairy product consumption and favorable changes in the gut microbiota composition, and these changes correlated with a reduced risk of cardiovascular disease development in mice and humans [80,81,82]. In addition, dairy products were found to affect immune and metabolic pathways [83]. A growing body of evidence presenting the association between dairy product consumption and gut microbiota composition and immunity, alongside the results found in the present study, suggest that the response to anti-PD-1 therapy may be affected by dairy product consumption. Yet, further studies, including a larger sample size and the heterogeneity of available dairy products, are warranted to better understand this aspect.

In our study, bowel movement frequency, but not stool type, was significantly associated with the response to anti-PD-1 therapy (Table 2). The majority of the responders and non-responders reported defecating once a day. However, significantly more of the non-responders reported frequently defecating every second day compared to the responders. Our results suggest that there is a correlation between gastrointestinal tract functioning and clinical responses to ICI treatment. This is in agreement with previous studies that demonstrated a negative correlation between bowel dysfunction (defined as constipation or laxative use) and clinical outcomes of ICI therapy in NSCLC and urothelial cancer patients [84,85]. Moreover, it was also indicated that stool consistency and frequency were associated with gut microbiota richness and composition [86,87]. For instance, one study demonstrated that individuals with a loose stool had lower microbial richness and higher abundance of the Prevotella enterotype compared to those with harder stools, whose fecal samples were enriched with the Ruminococcaceae-Bacteroides enterotype [86]. Another study demonstrated that there was a higher microbial richness and Bacteroidota to Firmicutes ratio in the individuals with a small number of defecations (≤2 times/week) than in those with a normal (1 time/day or 1 time/2 day) or large (≥2–3 times/day) number of defecations [87]. In our cohort, there were also revealed significant differences in the gut microbiota composition between the study groups (Figure 5). Moreover, there was a significant difference in the bowel movement frequency between the patients and controls, and the latter defecated more frequently, twice or more per day (Table 2).

Our study had several limitations. Firstly, the control group consisted of a small number of individuals, and the controls were significantly younger than the patients (Table 1). Moreover, several significant differences in terms of birthplace, plant portion consumption, and defecation frequency were found between the patients and controls (Table 2). These discrepancies between the study groups were taken into consideration during the analysis of the gut microbiota composition, as these factors were reported to affect the microbial composition in the intestines. Besides, the results from the questionnaire-based analysis led us to the conclusion that several aspects should be more specifically investigated. For instance, we found a correlation between dietary fat type consumption and the response to anti-PD-1 therapy (Table 2). However, we cannot indicate which type of dietary fat (or products) is associated with improved clinical outcomes, as the responders and non-responders differed significantly in terms of mixed dietary fat type consumption, and, additionally, there is a broad range of products that would be classified as a plant- or animal-based fats, which has a different impact on human health. Further studies investigating the impact of extrinsic factors modifying the gut microbiota and/or immune response, such as diet or medication use, should be more specified to better understand this complex interaction.

## 5. Conclusions

Based on our results presented in this paper and the published papers of other groups, we can hypothesize that a therapeutic intervention targeted for immunologic signaling can be modulated by different members of the gut microbiota, leading to the same therapeutic outcome. Gut-microbiota-specific members of distinct populations interplay with the same inflammatory/immunologic signaling pathways by different metabolites and/or nodes. Therefore, a personalized or at least population-based approach is more rational rather than a globalized undertake in modulating gut microbiota, for the improvement of ICI immunotherapy.

Further studies investigating the correlation between dietary patterns and the response to immunotherapy are needed. However, our results demonstrated that several dietary recommendations may be introduced before anti-PD-1 therapy commencement, to potentially improve clinical outcomes.

## Figures and Tables

**Table 1:** Baseline characteristics of all ICI patients and healthy subjects (*n* = 64).

Subjects Characteristics	Responders (R; *n* = 28)	Non-Responders (NR; *n* = 36)	*p* Value (R~NR)	Controls (C; *n* = 10)	*p* Value (C~R + NR)
Sex, *n* (%)			0.2 ^a^		0.732 ^a^
Male	15 (54)	25 (69)		5 (50)	
Female	13 (46)	11 (31)		5 (50)	
Age (years),			0.2 ^b^		0.0193 ^b^
median (range)	64 (41–84)	69 (32–92)		52.5 (36–67)	
M-stage at diagnosis ^c^, *n* (%)			0.3 ^a^	NA	NA
IV M1a	7 (25)	9 (23)			
IV M1b	5 (18)	5 (14)			
IV M1c	9 (32)	13 (36)			
IV M1d	4 (14)	9 (25)			
IIIc	3 (11)	0 (0)			
Serum LDH, *n* (%)			0.1 ^a^	NA	NA
Normal					
(≤250 U/L)	22 (79)	21 (58)			
Elevated					
(>250 U/L)	6 (21)	15 (42)			
Serum LDH,			0.04 ^b^	NA	NA
median (range)	197.5 (121–474)	238 (141–1173)			

LDH, lactate dehydrogenase. ^a^ Fisher’s exact test. ^b^ Kruskal–Wallis test. ^c^ American Joint Committee on Cancer (AJCC) 8th edition.

**Table 2:** Summary of the socio-demographic and dietary characteristics of study participants.

Demography	Responders (R, *n* = 25)	Non-Responders (NR, *n* = 32)	Controls (C, *n* = 10)	*p* Value (R~NR)	*p* Value (C~R + NR)	Clinical Benefit (CB, *n* = 34)	No Clinical Benefit (NB, *n* = 23)	*p* Value (CB~NB)
Sex, (*n*)				0.163 ^a^	0.191 ^a^			0.401 ^a^
Male	14	24	5			21	17	
Female	11	8	5			13	6	
Age (years), median (range)	64 (40–84)	67.5 (32–92)	52.5 (36–67)	0.239 ^b^ 0.183 ^a^	0.0193 ^b^ 0.073 ^a^	63 (32–85)	70 (38–92)	0.163 ^b^ 0.578 ^a^
31–40	0	2	1			1	1	
41–50	4	0	2			4	0	
51–60	5	6	3			7	4	
61–70	7	7	4			9	5	
71–80	7	9	0			8	8	
>80	2	6	0			4	4	
BMI, median (range)	27.8 (20–34.9)	27.5 (17.5–56.2)	25.1 (19.1–29)	0.526 ^b^ 0.497 ^a^	0.273 ^b^ 0.194 ^a^	27.1 (20–41.9)	27.8 (17.5–56.2)	0.612 ^b^ 0.401 ^a^
Underweight (<18.5 kg/m^2^)	0	1	0			0	1	
Normal (18.5–24.9 kg/m^2^)	9	6	5			5	2	
Overweight (25.0–29.9 kg/m^2^)	9	13	5			6	2	
Obese (≥30 kg/m^2^)	7	10	0			22	17	
Blood group:				0.635 ^a^	0.407 ^a^			0.323 ^a^
O	6	3	1			6	3	
A	8	10	3			13	5	
AB	1	2	3			1	2	
B	6	9	2			7	8	
Blood Rh-type:				0.101 ^a^	0.127 ^a^			0.041 ^a^
“−”	3	9	4			4	8	
“+”	18	15	5			23	10	
Antibiotic treatment:				0.804 ^a^	0.455 ^a^			0.850 ^a^
During last 2 months	3	2	0			4	1	
2–6 months ago	6	6	0			8	4	
6–12 months ago	3	6	2			5	4	
Over 12 months ago	11	13	8			14	10	
PPI usage:				1.0 ^a^	0.889 ^a^			1.0 ^a^
No	20	26	8			27	19	
Occasionally	3	3	2			4	2	
Regularly	1	1	0			1	1	
Antacid usage:				0.687 ^a^	0.267 ^a^			1.0 ^a^
No	21	26	7			27	20	
Occasionally	2	4	3			4	2	
Regularly	0	0	0			1	0	
Oral NSAID usage:				0.837 ^a^	0.523 ^a^			0.767 ^a^
No	13	15	3			18	10	
Occasionally	11	12	7			13	10	
Regularly	1	3	0			2	2	
Low-dose ASA:				0.741 ^a^	0.825 ^a^			1.0 ^a^
No	21	24	9			27	18	
Yes	4	6	1			6	4	
Birth:				1.0 ^a^	0.907 ^a^			0.738 ^a^
Natural	24	29	9			32	21	
Caesarean section	0	1	0			0	1	
Birthplace:				0.264 ^a^	0.058 ^a^			0.172 ^a^
Home	9	14	3			12	11	
Hospital	16	12	7			20	8	
Breastfed:				1.0 ^a^	1.0 ^a^			1.0 ^a^
No	0	0	0			0	0	
Yes	19	22	6			23	23	
Breastfed duration (months):				0.785 ^a^ 0.670 ^a,h^	0.708 ^a^ 1.0 ^a,h^			0.829 ^a^ 0.547 ^a,h^
24	8	9	2			10	7	
1	8	6	4			8	6	
0.25	1	0	0			1	0	
0.1	0	1	0			0	1	
Prevailing dietary fat type:				0.033 ^a^	0.092 ^a^			0.064 ^a^
Plant-based	12	19	7			16	15	
Mixed	9	2	1			10	1	
Animal-based	4	8	2			7	5	
Meat portions consumed:				0.53 ^a^	0.391 ^a^			0.801 ^a^
1 or fewer weekly	5	4	4			5	4	
2–6 weekly	15	23	6			22	16	
1 or more daily	3	2	0			4	1	
Vegetable portions consumed:				0.705 ^a^	0.695 ^a^			0.523 ^a^
1 or fewer daily	6	5	1			6	5	
2–3 daily	13	19	5			18	14	
4–5 daily	6	6	4			9	3	
Over 5 daily	0	0	0			0	0	
Fruit portions consumed:				0.768 ^a^	0.948 ^a^			0.422 ^a^
1 or fewer daily	8	7	3			10	5	
2–3 daily	13	20	6			17	16	
4–5 daily	3	2	1			4	1	
Over 5 daily	1	1	0			2	0	
Plant portions consumed ^c^:				0.082 ^a^ 0.058 ^a,d^ 0.041 ^a,e^ 0.033 ^a,d,e^	0.190 ^a^ 0.132 ^a,d^ 0.041 ^a,e^ 0.032 ^a,d,e^			0.031 ^a^ 0.044 ^a,d^ 0.041 ^a,e^ 0.032 ^a,d,e^
Low	2	11	1			4	9	
Recommended	10	14	5			13	11	
High	7	5	3			10	2	
Fermented veg. consumption:				0.835 ^a^	0.815 ^a^ 0.403 ^a,d^			1.0 ^a^ 0.820 ^a,d^
No	3	2	0			3	2	
Rarely	6	8	4			8	6	
Often	16	20	6			22	14	
Salt consumption:				0.316 ^a^	0.596 ^a^			0.216 ^a^
Low	10	12	5			12	10	
Average	14	13	4			19	8	
High	1	5	1			2	4	
Dairy portions consumed:				0.072 ^a^ 0.050 ^a,g^	0.217 ^a^			0.037 ^a^ 0.024 ^a,g^
1 or fewer daily	14	8	4			17	5	
2–3 daily	10	19	5			13	16	
4–5 daily	1	1	1			1	1	
Over 5 daily	0	2	0			2	0	
Bread type consumption:				0.55 ^a^ 0.200 ^a,d^	0.744 ^a^ 0.507 ^a,d^			0.181 ^a^ 0.266 ^a,d^
Light only	2	5	1			4	3	
Mostly white	9	13	6			11	11	
Mostly wholemeal	11	8	2			15	4	
Wholemeal only	2	3	1			2	3	
Cereal consumption:				0.93 ^a^ 0.821 ^a,d^ 0.764 ^a,h^	0.866 ^a^ 0.581 ^a,d^			0.769 ^a^ 0.725 ^a,d^ 0.547 ^a,h^
Breakfast cereals, white rice	3	3	0			3	3	
Mostly listed above	6	5	3			7	4	
Mostly listed below	5	8	1			8	5	
Oatmeal, muesli, brown rice	9	9	4			13	5	
Beverage sweetening habits:				0.293 ^a^	0.366 ^a^ 0.194 ^a,d^			0.353 ^a^ 0.164 ^a,d^
Do not sweeten	12	20	7			18	14	
Use artificial sweetener	1	2	1			1	2	
Use sugar	12	8	2			14	6	
Soft drink consumption:				0.71 ^a^	0.469 ^a^ 0.446 ^a,d^			0.976 ^a^ 0.909 ^a,d^
1 or fewer servings/month	10	14	5			15	9	
2–3 servings/month	3	6	4			5	4	
1–3 servings/week	5	6	0			6	5	
More than 3 servings/week	5	3	1			5	3	
Defecation frequency:				0.01 ^a^	0.038 ^a^			0.720 ^a^
Twice or more per day	5	4	3			3	1	
Once a day	15	13	5			6	7	
Every second day	1	12	2			17	11	
Seldom	3	1	0			6	3	
Bristol Stool Form Scale:				0.689 ^a^	0.713 ^a^			0.747 ^a^
Type 1	1	5	2			3	3	
Type 2	2	2	0			3	1	
Type 3	3	6	2			9	3	
Type 4	13	14	5			15	12	
Type 5	2	2	0			2	2	
Type 6	0	0	0			0	0	
Type 7	0	0	1			0	0	
Diet alterations:				0.362 ^a^	0.182 ^a^			0.373 ^a^
No	23	25	8			30	18	
Yes, during last 2 weeks	1	4	1			0	0	
Yes, during last month	0	0	1			0	0	
Yes, over 1 month ago	0	0	0			2	3	
Current probiotics use:				1.0 ^a^	1.0 ^a^			0.362 ^a^
No	21	23	9			28	16	
Yes	2	3	1			2	3	
Probiotic use history:				0.649 ^a^	0.740 ^a^			0.218 ^a^
In last 2–3 weeks	0	1	1			0	1	
Over 3 weeks ago	1	2	0			3	0	
In last 6 months	1	2	1			2	1	
Over 6 months ago	3	1	3			4	0	
Tobacco smoking:				1.0 ^a^	0.494 ^a^			1.0 ^a^
No	20	26	7			27	19	
Yes	4	4	3			5	3	
Smoking cessation:				1.0 ^a^	0.339 ^a^			0.251 ^a^
Less than 1 year ago	0	1	0			0	1	
1 to 2 years ago	1	1	1			2	0	
Over 2 years ago	7	8	3			10	5	
Alcohol consumption history:				0.253 ^a^	0.430 ^a^			0.740 ^a^
Never	3	5	1			5	3	
In the past	8	12	3			11	9	
Currently	14	8	6			15	7	
Alcohol consumption freq. ^f^:				0.495 ^a^	0.748 ^a^			0.491 ^a^
1 or fewer servings/month	6	1	0			6	1	
2–4 servings/month	5	4	3			6	3	
1–6 servings/week	1	2	2			1	2	
1–2 servings/day	1	1	0			1	1	
3 or more servings/day	1	0	0			1	0	

NSAID, non-steroidal anti-inflammatory drugs; PPI, proton pump inhibitor. ^a^ Fisher’s exact test. ^b^ Kruskal–Wallis test. ^c^ Pooled categories of vegetable and fruit portions. ^d^ Only those that declared no diet change. ^e^ For low versus high. ^f^ In group declaring present alcohol consumption. ^g^ For ‘1 or fewer servings daily’ versus ‘2–3 servings daily’. ^h^ Collapsed to 2 categories.

**Table 3:** Bacterial ASVs significantly enriched in responders (log_2_FC positive values) and non-responders (log_2_FC negative values).

	log2FC	*p*-Value	FDR *p*-Value	Phylum Class	Order Family	Genus Species
ASV621	−24.39	3.29 × 10^−28^	6.24 × 10^−25^	Firmicutes Bacilli	Izemoplasmatales NA	NA NA
ASV865	−23.52	9.94 × 10^−23^	9.41 × 10^−20^	Firmicutes Clostridia	Oscillospirales Oscillospiraceae	NA NA
ASV147	−23.73	3.19 × 10^−20^	2.01 × 10^−17^	Firmicutes Clostridia	Clostridia UCG-014 NA	NA NA
ASV338	−25.25	1.29 × 10^−17^	6.11 × 10^−15^	Firmicutes Clostridia	Clostridia UCG-014 NA	NA NA
ASV166	−23.70	1.06 × 10^−15^	4.03 × 10^−13^	Firmicutes Clostridia	Oscillospirales Ruminococcaceae	*Faecalibacterium prausnitzii*
ASV85	23.48	1.62 × 10^−15^	4.89 × 10^−13^	Bacteroidota Bacteroidia	Bacteroidales Prevotellaceae	*Prevotella* *copri*
ASV112	23.44	1.81 × 10^−15^	4.89 × 10^−13^	Bacteroidota Bacteroidia	Bacteroidales Prevotellaceae	*Prevotella* *copri*
ASV394	22.61	1.69 × 10^−14^	4.00 × 10^−12^	Bacteroidota Bacteroidia	Bacteroidales Bacteroidaceae	*Bacteroides* *uniformis*
ASV809	−22.22	5.49 × 10^−14^	1.15 × 10^−11^	Desulfobacterota Desulfovibrionia	Desulfovibrionales Desulfovibrionaceae	*Desulfovibrio intestinalis*

## Data Availability

The raw data are available in the RepOD repository (https://doi.org/10.18150/6F2OSE, accessed on 29 August 2022). Other datasets generated during and/or analyzed during the current study are available from the corresponding author upon reasonable request.

## Supplementary Materials

The following supporting information can be downloaded at: https://www.mdpi.com/article/10.3390/cancers14215369/s1. Document S1: Questionnaire; Figure S1: Whole-genome phylogenetic tree of a selected subset of the four *P. copri* clades comprising the *P. copri* complex in relation to reconstructed *Prevotellaceae* metagenome-assembled genomes. *P. copri* clade A members form primary cluster 10, clade B—primary cluster 9, clade C—secondary clusters 11_6 to 11_10, clade D—secondary clusters 11_1 to 11_5, and other members of the genus *Prevotella* and *Prevotellaceae* family were assigned to primary clusters 1–8 and 12–16; Table S1: Summary of shotgun metagenomic sequencing and assembly statistics; Table S2: Publicly available datasets utilized and the *Prevotellaceae* reconstructed MAGs statistics. List of the selected 20 exemplars, curated *P. copri* MAGs belonging to four distinct clades, and 19 *Prevotellaceae* MAGs reconstructed from shotgun metagenome sequencing of the stool microbiome with their associated genome statistics.
