# Supplementary material for: A Clinical Outcome of the Anti-PD-1 Therapy of Melanoma in Polish Patients Is Mediated by Population-Specific Gut Microbiome Composition
Source: Cancers (Basel). 2022 Oct 31;14(21):5369. doi: 10.3390/cancers14215369 (PMC9653730; doi:10.3390/cancers14215369)
Supplement: Supplementary file 1 [file cancers-14-05369-s001.zip › Document S1.pdf]

The questionnaire (translated version)

1. Today's date:
2. Year of birth
3. Place of birth:
4. Height: ……………cm.
5. Body mass: …………………kg.
6. Sex:
    a. M
    b. F
7. Birth:
    a. natural
    b. Caesarean section
    c. don't know
8. Birthplace:
    a. Hospital
    b. Home
    c. don't know
9. Breastfed:
    a. yes
    b. no
    c. don't know

If yes, how long have you been breastfed?

a. <1 week   b. >1 week   c. >1 month   d. >1 year

10. Blood group:
    a. A+ b. A- c. B+ d. B- e. O+ f. O- g. AB+ h. AB-
11. Only for women: are you pregnant?:
    a. yes
    b. no
12. Only for women: do you have a regular menstrual cycle?:
    a. yes
    b. no
13. Are you smoking tobacco?:
    a. Yes
    b. No
    c. I ceased smoking tobacco ……………………………….. ago.
14. Do you have a diagnosed gastrointestinal tract disease (you can choose more than one answer):
    a. Barrett's esophagus
    b. Celiac disease
    c. Cancer
        i. Colon
        ii. Stomach
        iii. Esophagus
        iv. Pancreas

v. Liver

d. Diverticular disease
e. Duodenal ulcer
f. Indigestion
g. Stomach ulcer
h. Gastroesophageal reflux disease
i. Viral hepatitis
j. *H. pylori* infection
k. inflammatory bowel disease
l. Leśniowski-Crohn disease
m. ulcerative colitis
n. irritable bowel syndrome
o. Lactose intolerance
p. Liver disease
q. Acute pancreatitis
r. Other diseases: ………………………………….

15. Did you have any diagnostic test or treatment of gastrointestinal tract within the last one month?

a. Yes
b. No

If yes, which one?

a. rectoscopy b. colonoscopy c. other: ……………………………………………………….

16. When did you use antibiotics for the last time:

a. Within last 2 months
b. 2-6 months ago
c. 6-12 months ago
d. More than 12 months ago

17. Bristol Stool Form Scale:

a. Type 1: Separate hard lumps, like nuts (hard to pass)
b. Type 2:Sausage-shaped but lumpy
c. Type 3: Like a sausage but with cracks on its surface
d. Type 4: Like a sausage or snake, smooth and soft
e. Type 5: Soft blobs with clear-cut edges (passed easily)
f. Type 6: Fluffy pieces with ragged edges, a mushy stool
g. Type 7: Watery, no solid pieces, entirely liquid

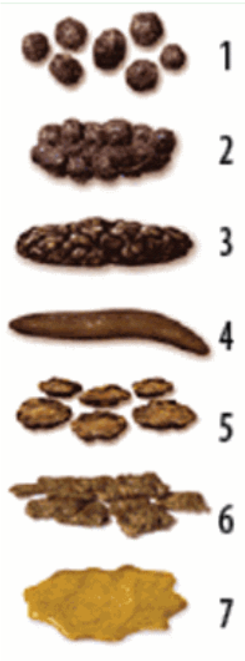

18. Defecation frequency:

a. >2 time a day
b. Once a day
c. Once every second day
d. Seldom

19. Current diet:

a. Diversified
b. Vegetarian
c. Vegan
d. Other (e.g. diabetes) …………………………………………….

20. Did you change your diet in the last time:
    a. No
    b. Yes, within last 2 weeks
    c. Yes, within last 2 month
    d. Yes, over 1 month ago
21. Do you consume alcoholic beverages (e.g. beer, wine, liquor, etc.)?:
    Yes No, but I used to consume No, I have never consumed
    If yes, what is your average consumption of alcoholic beverages (1 serving is about 0.5l of beer, a glass of wine - about 200 ml, a glass of vodka or other high alcohol - about 25 ml):

    Less than 1 serving per month
    1-4 servings per month
    1-6 servings per week
    1-2 servings daily
    3 or more servings per day

22. Are you currently taking probiotic preparations: Yes No
    if you are not currently taking them, but in the past you did - when did you last take them:
    within the last 2-3 weeks
    over 3 weeks ago
    in the last 6 months
    over 6 months ago
23. Do you consume fermented dairy drinks (e.g., yogurt, kefir):

    Yes. No

24. What type of fats do you mostly use to prepare your meals:
    Vegetable (margarine, olive oil, oil)
    animal (butter, lard)
25. How many servings of meat do you consume on average (1 serving is about 150g which is about ½ chicken breast, chicken leg, 2-3 thin sausages):
    1 or less than 1 - per week
    2-6 per week
    1-2 daily
    more than 2 daily
26. On average, how many servings of vegetables do you eat per day (1 serving is a large potato, bell pepper, tomato, a glass of coleslaw, 5 leaves of lettuce, a glass of vegetable juice):
    less than 1
    2-3
    4-5
    more than 5
27. On average, how many servings of fruit do you consume per day (1 serving is a medium apple, pear, 2 plums, banana, orange, a handful of grapes or other small fruits, a glass of juice):
    less than 1
    2-3

4-5
more than 5

28. On average, how many servings of milk and dairy products do you consume per day (1 serving is a glass of milk or yogurt, ¾ cup of thick yogurt, a medium slice of cottage cheese, a small processed cheese, 2 slices of yellow cheese):
less than 1
2-3
4-5
więcej niż 5
29. What kind of bread do you usually eat:
only light (wheat)
mostly light, rarely dark
mostly dark, less often light
dark only (wholemeal or graham type)
30. What kind of grain products do you usually eat:
a) Only sweetened breakfast cereals (chocapic or cheerios type), white rice, semolina
b) mostly products listed in a), less often those listed in d)
c) mostly products listed in d), less often those listed in a)
d) Only cereals (such as oatmeal), muesli, groats (such as buckwheat, barley), brown rice
31. Do you sweeten your tea, coffee or other beverages?
Yes, with sugar (white, brown), honey or syrup
Yes, with sweetener (including xylitol)
I don't sweeten
32. How often do you drink sweetened juices or sodas:
Less than once a month
2-3 times a month
1-3 times a week
More than 3 times a week
33. Are you using proton pump inhibitors (e.g., Ortanol, Controloc control, Polprazol) for gastrointestinal reflux (aka heartburn):
Yes, on a regular basis
Yes, on an ad hoc/auxiliary basis
No
34. Are you using gastric juice acid neutralizers (e.g. Rennie, Manti, Gealcid) for gastrointestinal reflux (aka heartburn):
Yes, on a regular basis
Yes, on an ad hoc/auxiliary basis
No
35. Do you use acetylsalicylic acid (aspirin) in low doses (75-150mg, e.g. Polocard, Aspirin Cardio) for the prevention of cardiovascular disease and other diseases involving clots in the vessels:
Yes
No
36. Do you use non-steroidal anti-inflammatory drugs (e.g. aspirin, polopyrin, paracetamol, ibuprom) to relieve chronic pain:
Yes, more than 3-4 times a month

Yes, rarely
No
37. Do you consume fermented vegetable products (pickles, sauerkraut):
    Yes, more than 3-4 times a month
    Yes, rarely
    No
38. Do you prefer food that is "well" salted:
    Yes, I often salt my dishes
    I think my salt intake is at an average level
    No. I take care to keep my consumption of table salt low
