# Supplementary figures and images for: A Clinical Outcome of the Anti-PD-1 Therapy of Melanoma in Polish Patients Is Mediated by Population-Specific Gut Microbiome Composition
Source: Cancers (Basel). 2022 Oct 31;14(21):5369. doi: 10.3390/cancers14215369 (PMC9653730; doi:10.3390/cancers14215369)

MASH clustering

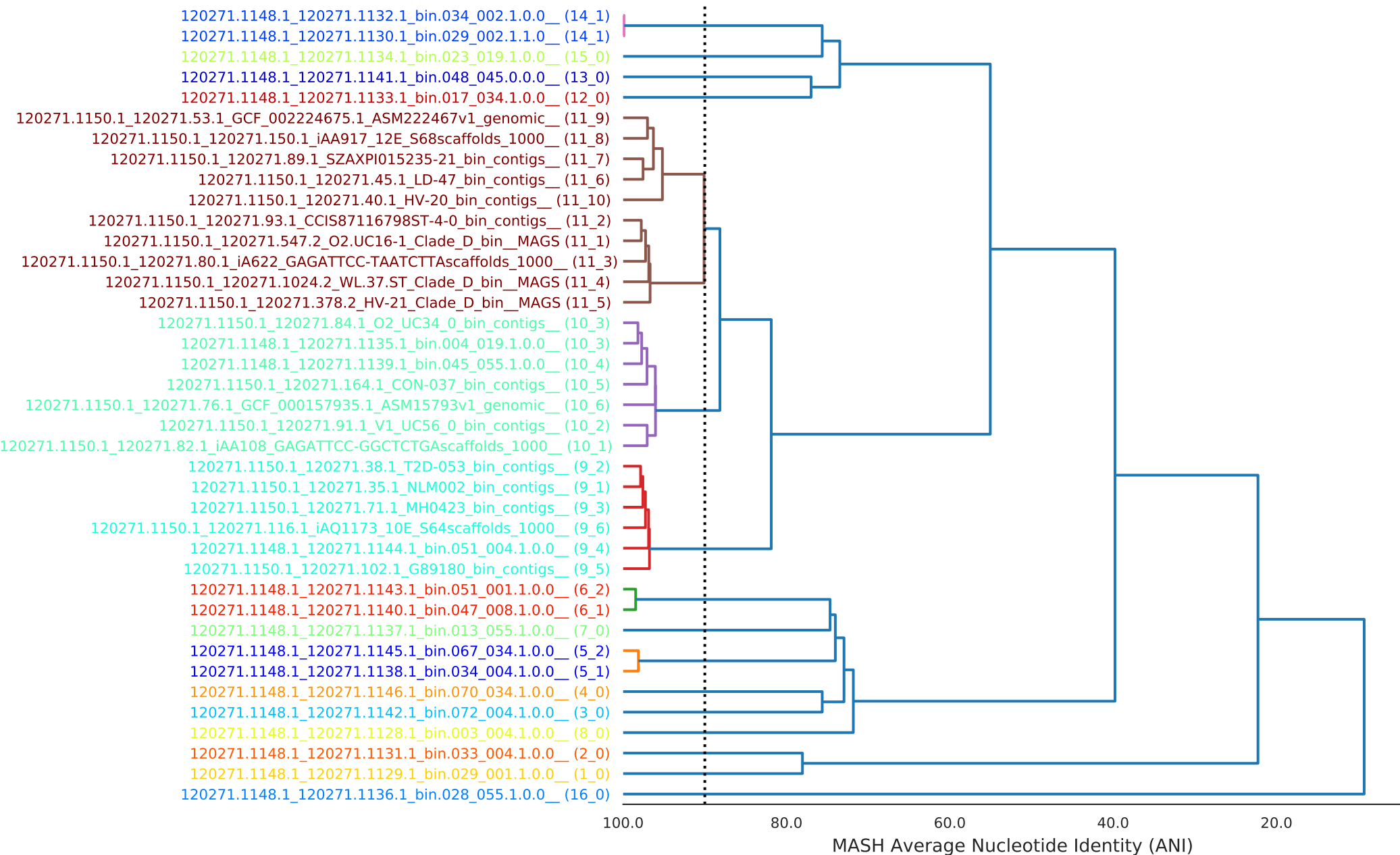

Supplement: Supplementary file 1 [file cancers-14-05369-s001.zip › Figure S1_Clustering_dendrogram.pdf]
